# Short-term propofol infusion syndrome (PRIS): fact or fiction? A systematic review on early PRIS in intensive care and anesthesia

**DOI:** 10.1186/cc14560

**Published:** 2015-03-16

**Authors:** J Vandenbrande

**Affiliations:** 1 University Hospitals Leuven, Belgium

## Introduction

Propofol infusion syndrome (PRIS) is a rare propofol complication, leading to cardiac failure. It was first described in critically ill children and in adults with traumatic brain injury. Pathophysiology is unknown although common factors are the prolonged (>48 hours) use of high-dose (>5 mg/kg/hour) propofol combined with elevated levels of catecholamines and corticosteroids. Recently, case reports of early-onset PRIS during anesthesia and in the early postoperative setting were published. In many of these, lactic acidosis is interpreted as onset of PRIS. Criticism offers that it might concern a poor differential diagnostic approach or an observational bias. Also, lactic acidosis is not an obligate PRIS symptom and incidence of lactic acidosis during propofol sedation is unknown. To gain insight into the incidence and characteristics of early PRIS, we performed a systematic review on early PRIS cases.

## Methods

A literature study via MEDLINE and Embase search with keywords 'PRIS', 'lactic acid', 'propofol' and 'sedation'. All cases in English, French and Spanish were indentified. Exclusion criteria were onset >48 hours, unclear description of time pattern and dose.

## Results

Twenty-two cases of early PRIS were found. These concern 10 pediatric versus 12 adult patients. Eleven were identified in the ICU versus 11 in the operating room. The survival rate of early PRIS was 95.5%, and morbidity was restricted to four patients. In the adult subgroup, the mean propofol dose was 4.9 mg/kg/hour. Triggering factors such as use of catecholamines and corticosteroids were found in 36.4% and 45% of patients. In total, 3/22 cases match Bray's definition of PRIS. In 14/22 cases, lactic acidosis was interpreted as onset of PRIS.

## Conclusion

Compared with a review by Fudickar [1], we found significant differences in critical dose, risk factors, symptomatology and morbidity/mortality between PRIS and early PRIS cases. As criticisms are offered, a question is whether these cases really are the onset of the fatal syndrome PRIS. Therefore, we completed differential diagnosis of lactic acidosis and found that not all possible causes (for example, hyperglycemia, ketonemia, pharmacologic confounders as biguanides, epinephrine) were ruled out in most cases. This is important since PRIS is an exclusion diagnosis. The existence of early PRIS should indeed be questioned and investigated by large, multicenter observational trials.

## References

[B1] Fudickar A Propofol infusion syndrome in anaesthesia and intensive care medicine Curr Opin Anaesthesiol 2006 19 404 10 16829722 10.1097/01.aco.0000236140.08228.f1

